# Supplementary material for: Are carbon emissions trading and green financial instruments synergistic? -Comprehensive quantitative research based on content analysis
Source: PLoS One. 2024 Mar 7;19(3):e0298601. doi: 10.1371/journal.pone.0298601 (PMC10919601; doi:10.1371/journal.pone.0298601)
Supplement: S1 Data — (ZIP) [file pone.0298601.s001.zip › Data/地级市Z_F工作报告.zip!/╣π╢1⁄2/╒╪╟∞╩╨/└┤╫╘╣1⁄2╓┌║┼í╛┬φ┐╦╩2╛▌═°í┐_2019.docx]

各位代表：

　　现在，我代表肇庆市人民政府向大会作工作报告，请予审议，并请政协委员和其他列席人员提出意见。

2018年工作回顾

　　过去一年，市政府坚持以习近平新时代中国特色社会主义思想为指导，全面贯彻党的十九大和十九届二中、三中全会精神，认真学习贯彻习近平总书记对广东重要讲话和重要指示批示精神，全面落实省委省政府决策部署和省委书记李希同志肇庆调研讲话要求，按照省委“1＋1＋9”工作部署，在市委的正确领导下，在市人大和市政协的监督支持下，坚持稳中求进工作总基调，贯彻新发展理念，深化供给侧结构性改革，解放思想、东融西联、产业强市、实干兴肇，聚焦新区崛起府城复兴，加快推进产业、乡村、旅游三大振兴，深入开展全国文明城市、国家创新型城市、国家生态文明建设示范市三大创建，坚决打好三大攻坚战，全力以赴稳增长、促改革、调结构、惠民生、防风险，经济社会健康可持续发展，市十三届人大四次会议确定的目标任务基本完成。

　　一、经济进一步呈现稳中向好态势

　　较好完成预定主要目标。预计（下同）完成地区生产总值2201.8亿元，比上年增长6.6%（以下简称“增长”）。一般公共预算收入106.04亿元，增长11.8%，增速居全省第2位，非税收入占比22.7%、下降5.2个百分点。规模以上工业增加值增长7.9%。固定资产投资1343.01亿元，增长10%；100项市重点项目年度计划投资完成率118%。社会消费品零售总额增长10.2%。外贸进出口总额增长8.9%。全体居民人均可支配收入2.43万元，实际增长6.6%。

　　实体经济稳步提升。修订“实体经济十条”，建立“书记市长强企直通车”等面对面解决企业困难的常态化机制，多措并举为企业减负超43亿元。兑现“366”“1133”工程奖励超2亿元，三大主导产业占规上工业总产值比重由15.6%提升到30.2%，新增年主营业务收入超亿元工业企业36家，高新区宏旺金属年主营业务收入突破百亿元，实现超百亿工业企业零的突破。新增规模以上工业企业155家，新增工业技改企业286家，工业技改投资增长18.9%。引进发展金融机构6家，新增区域性股权交易市场挂牌企业10家。

　　重大风险防范化解有效可控。坚持大融资大建设大开发推动城市价值大提升，制定实施隐性债务风险化解方案，获得省各类补助资金和债券资金212亿元，政府债务总体稳定可控，大融资效应逐步显现。全面完成农合机构改制农商行申筹工作，怀集、广宁农商行开业运作。全市金融机构本外币各项存款余额增长10.5%，贷款余额增长21.5%、在全省排第3位，不良贷款率2014年以来首次低于全省平均水平。房地产市场保持平稳发展。

　　二、工业发展“366”工程步伐加快

　　“招商引资年”实现预期目标。成功举办2018“肇庆金秋”、中国环境上市公司峰会、中国民商论坛、中国工业论坛等高端经贸交流活动和深圳、北京、杭州等招商推介活动。新引进产业合同项目219宗，计划投资总额2060亿元，国信通等6个超百亿项目签约落户，华侨城项目实现当年引进当年动工。

　　“园区建设年”进展顺利。新开发及盘活工业园区用地10.3平方公里，新建成及盘活改造通用厂房181万平方米，其中科技孵化器面积91万平方米。承接珠三角产业梯度转移项目74个，计划投资总额363亿元。高要、广宁、封开产业转移集聚地被认定为省级产业转移工业园。

　　“项目落地年”成效明显。实施产业投资项目直接落地“双容双承诺”制度，推动总投资419亿元的30个产业项目直接落地。小鹏、鼎星等新能源汽车项目建设加快，新开工新投产超亿元先进装备制造项目33个，德通环保设备等节能环保产业项目顺利投产。100个计划新增落地开工项目全部动工，年度投资完成率118.6%。57个计划竣工项目实现投产。

　　三、创新驱动发展“1133”工程取得新进展

　　发展新动能进一步增强。高新技术企业净增124家，总数达到413家，增长43%，309家企业通过国家科技型中小企业评价入库。肇庆学院成为硕士学位授予立项建设单位，广东工商职业学院成功升本，广东华商学院、华南农业大学珠江学院、广东健康医学院（筹）新校区动工建设，广东华航航空学院签约落户。新增省级新型研发机构2家、创新平台33家，规上工业企业研发机构覆盖率超40%。新增科技企业孵化器15家、众创空间4家。完成68家国有“僵尸企业”出清重组、419家传统低效产业企业退出和整治提升工作。

　　肇庆高新区创新能力持续提高。列入珠三角国家科技成果转移转化示范区，珠三角国家自主创新示范区、国家知识产权示范园区加快建设，高新技术企业增加到116家。哈尔滨工业大学新能源汽车轻量化复合材料工程技术研究院、武汉大学粤港澳环境技术研究院等新型研发机构进驻运行，国腾量子公司获批省重大科技专项。新签投资协议项目58宗、计划投资总额超500亿元，浩宇机械等28个项目开工建设，唯品会二期等23个项目投（试）产，纳税超千万元企业达58家。

　　高层次人才引育力度加大。深入实施“西江人才计划”，成功举办2018亚太地区可持续发展论坛暨首届肇庆人才节。引育国家“万人计划”专家2名，柔性引进长江学者等国家级高层次人才25名，引进第二批西江创新团队8个、领军人才6名，肇庆学院引进百名港澳台博士，为124名高层次人才颁发“人才绿卡”，实现重大人才项目工程引育“全覆盖”。

　　四、改革开放注入新动力

　　积极融入粤港澳大湾区建设。研究起草粤港澳大湾区实施方案和行动计划，主动开展政府间合作交流，谋划建设大湾区具有重大影响力的合作平台。加快构建1小时交通圈，肇庆到深圳高铁班次增加到每天4对8趟，汕昆高速肇庆段建成通车，汕湛高速、怀阳高速、广佛肇高速二期大旺至广州石井（肇庆段）加快建设。肇庆（怀集）绿色农副产品集散基地纳入国家战略。

　　“数字政府”改革走在全省前列。“数字政府”综合改革试点取得全省“六个第一”，初步实现“一门受理、一网通办、一次办成”。政务服务网全面使用，统一实名身份认证，314项事项“零跑动”办理，980项事项“一次办成”。不动产交易登记最快3天出证。开办企业便利度连续两年位居全省第4，新登记各类企业1.1万户，增长44%。电子政务服务能力指数排在全省第4。

　　“放管服”改革力度加大。取消调整市级行政职权720项，确保高要区有完整的县级行政职权，继续向端州和高新区委托或下放行政职权。深化投资项目建设审批制度改革，在16个面向群众、企业办事多的单位设立行政审批科，企业、政府投资项目审批时间分别压缩到29和32个工作日，图审市场化改革走在全省前列。深入推进“减证便民”，取消各类证明事项158项。扩建升级市行政服务中心，办理事项扩展到50个部门760多项。政府公共服务满意度排名全省第4。

　　五、高品质新都市建设进入快车道

　　三年省运筹备圆满收官。成功举办第十五届省运会和第八届省残运会，展现新时代新肇庆新风貌，我市代表团金牌数、奖牌数和团体总分均位列全省第3。如期完成34个体育场馆建设改造任务，实现县（市、区）场、馆全覆盖。肇庆火车站、肇庆东站综合体投入运营，东进大道一二期、江滨堤路、紫云大道、鼎湖大道等建成通车，省运承办与城市发展取得双丰收，增强了肇庆自信，凝聚了肇庆力量。

　　新区崛起府城复兴加快城市扩容提质。肇庆新区累计完成投资338亿元，65平方公里先行区主要骨架路网初步建成，18平方公里起步区城市框架基本形成，8平方公里核心区基本成型。建成城市干道53公里、地下综合管廊26.5公里、城市绿地1500亩。体育中心运营使用，安置小区康乐花园交付入住，医院、学校、酒店、商务中心等公共服务设施配套进一步完善，京东华南运营中心等一批计划总投资近770亿元优质产业项目签约落户，标志性工程“西江明珠”动工建设。府城保护与复兴项目累计投入近15亿元，基本完成府城二期房屋征拆、包公府衙片区考古调查勘探，府城公园一期建成开放，相关规划设计进一步优化提升。

　　精准创文推动城市管理上新台阶。坚持创文为民惠民，实施“八大整治提升行动”，建成一批街头小景，整治一批背街小巷和城市出入口，清拆户外广告超17万平方米，绿化美化净化亮化彩化工程成效明显。端州城区50条“瓶颈路”完成征拆44条、通车32条。推进智能化交通治理，成为全省交通最畅通城市之一。党政机关和事业单位停车场、卫生间全面向社会开放。建成“一市四区”数字化城市管理平台，推进“市县镇村”四级联创，四会全国文明城市创建成果不断巩固，德庆开展全国县级文明城市创建，广宁、封开、怀集创建省县级文明城市，形成上下一心同创文的“大合唱”格局。

　　旅游振兴势头初步形成。成立市旅游发展委员会，编制全域旅游发展规划，全力建设彰显中国特色、展示岭南文化的世界旅游目的地。星湖景区创5A通过省专家组初审并报国家景评委。竞演《魅力中国城》，获得“优秀魅力城市”等荣誉，“中国砚都·七星肇庆”品牌进一步提升。举办肇庆命名900周年国际马拉松赛、“迎省运·肇庆命名900周年”精品文物展、第三届全球华人少年书法大会总决赛、粤港澳大湾区（肇庆）光影艺术节、粤港澳大湾区旅游美食嘉年华、全国体操锦标赛、全国田径大奖赛等重大活动，赛事经济和文旅产业融合发展，推动旅游总收入增长6%。

　　六、乡村振兴开局良好

　　精准脱贫攻坚取得阶段性进展。制定实施精准脱贫三年行动方案，全年投入扶贫资金2.94亿元，再实现1.4万贫困人口预脱贫，贫困发生率下降到0.35%。积极开展对口桂林、贺州两市四县扶贫协作。

　　规划并实施现代农业发展“611”工程。获批高要南药、德庆贡柑、广宁油茶、怀集丝苗米4个省级现代农业产业园，新增一批农业龙头企业、农民合作示范社、示范性家庭农场，粮食产量持续增长。新增省农业名牌产品9个、农产品地理标志2个。创建省级休闲农业与乡村旅游示范镇3个、示范点9个，高要“河乐水”等乡村旅游示范区建设扎实推进。互联网+现代农业平台落地见效。基本完成农村土地承包经营权确权登记颁证。

　　农村人居环境综合整治全域推进。“三清三拆三整治”进展顺利，四会、德庆全域推进农村人居环境整治示范县（市）建设。“村村通”自来水工程全面完成，农村生活垃圾处理率达97.6%，农村公路安防工程建成4316公里，“四好农村路”完成省建设任务。7个乡村振兴示范镇“书记项目”全面启动，5个省级新农村连片示范工程有力推进，省际接壤地区乡村振兴示范带启动建设。111个省定贫困村全部达到干净整洁村标准。

　　七、民生事业持续进步

　　污染防治攻坚战扎实开展。认真抓好中央环保督察“回头看”和省环保督察反馈问题整改。狠抓空气污染五大源头整治，城区空气质量进一步改善，综合指数同比下降3.4%。全面落实各级河长制湖长制，排查治理北岭山违法用水和星湖周边141个污染源，推动星湖水质持续改善。羚山涌、石咀涌黑臭水体有效整治，鼎湖、四会违法禽畜养殖场清理基本完成，水质综合指数位列全省第2。固体废物非法处置专项整治全面开展。实施瓷土矿资源开发秩序专项整治，不新批采矿权，27个到期矿权全部停产，复垦复绿面积1100多亩。肇庆环保能源发电项目动工建设。造林13.7万亩，建成3个省级森林小镇。国家生态文明建设示范市创建指标完成率61.1%。

　　发展成果更多惠及人民群众。财政投入民生类支出228亿元，占一般公共预算支出72.4%。全面完成年度民生实事。城镇新增就业4.4万人。成为省推进教育现代化先进市、省现代职业教育综合改革示范试点市，新建、扩建义务教育学校19所，新增义务教育学位2万多个。市妇幼保建院综合楼等项目加快推进，新增床位3600张，组建医联体21个。创建省卫生镇4个、省卫生村397个，农村卫生厕所普及率达到99%。城乡居民基本养老保险和基本医疗保险覆盖率均超过99%。棚户区改造住房建成3901套，累计分配公租房10217套。新建社区体育公园10个，行政村（社区）综合性文化服务中心实现全覆盖。公共交通“四大畅通工程”加快推进，新开通及调整延伸公交线路19条、开通定制线路35条，新增纯电动公交车356辆。全面推进扫黑除恶专项行动，打掉涉黑涉恶团伙169个，“两抢一盗”犯罪案件同比下降19.6%，“智慧新指挥”成为全省示范点，人民群众安全感更加充实。安全生产形势、食品药品安全和社会治安大局总体稳定。国防教育、国防动员、军民融合和“双拥”工作不断加强。完成与鸡西市对口合作年度任务。统计、民族、宗教、革命老区、外事、侨务、港澳、对台、人防、地方志、信访、档案、气象、水文、地震、妇女儿童、青少年、老龄、残疾人、慈善等工作取得新成效。

　　八、政府自身建设不断加强

　　全面落实从严治党主体责任，执行“第一议题”制度，认真开展“大学习、深调研、真落实”，促进全市政府系统树牢“四个意识”，坚定“四个自信”，坚决做到“两个维护”，全面彻底肃清李嘉、万庆良恶劣影响。坚持向市人大及其常委会报告工作，向市政协通报情况，按期办结人大代表建议120件、政协提案163件。开通人大代表、政协委员“市长直通车”，办结建议24件。组织起草市人大地方性法规草案3件，出台市政府规范性文件39件，行政复议案件依期结案率100%。严格执行中央八项规定及其实施细则精神，推行土地不招拍挂不改功能、不增加容积率、建设类项目不使用EPC模式、不开新矿的“四不”廉政风险防控机制，实行政府投资性工程审计调查前移和随机抽查等监督机制，严控工程造价概算和预算。实行“谁牵头、谁负责；谁配合、谁协助”机制和“一线工作法”、重点工作会商制，全面推进无纸化政务系统应用，政府服务效能进一步提升。认真抓好中央、省委巡视和国务院大督查等发现问题的整改落实，有效解决一批突出问题。

　　各位代表，这些成绩来之不易，这是在习近平新时代中国特色社会主义思想的引领下，在省委、省政府和市委的坚强领导下，在市人大、市政协的监督支持下，全市干部和全市人民以及社会各界开拓进取、务实拼搏、凝心聚力、团结奋斗的结果。我代表市人民政府，向全市人民，向各位人大代表、政协委员，向各民主党派、各人民团体，向驻肇部队和单位，向老领导、老同志，向所有关心支持参与肇庆现代化建设的海内外各界人士，致以崇高的敬意和衷心的感谢！

　　各位代表，我们也清醒地认识到，我市经济社会发展还存在不少困难和问题。比如：产业结构还需进一步优化，新旧动能转换相对偏慢，陶瓷等传统产业转型升级压力依然较大，成效不够明显；“三个年”活动还需进一步推进，尤其是产业落地动工还需要下决心解决用地难、融资难等痛点、难点、堵点问题；空气质量在全省排名依然靠后，国家生态文明建设示范市创建离达标仍有差距；农村一二三产融合度不高，城乡区域发展差距依然较大，解决发展不平衡不充分问题的硬招实招还不多；政府自身建设仍需加强，离人民群众对满意政府的要求仍有差距。对这些问题，我们要采取坚决有力措施加以解决。

2019年工作安排

　　2019年是新中国成立70周年，是全面建成小康社会关键之年，做好今年工作极为重要，意义非凡。我们要以改革开放眼光看待改革开放，以新担当新作为奋力开创肇庆工作新局面。

　　政府工作的总体要求是：高举习近平新时代中国特色社会主义思想伟大旗帜，全面贯彻党的十九大和十九届二中、三中全会以及中央经济工作会议精神，全面贯彻落实习近平总书记对广东重要讲话和重要指示批示精神，认真贯彻落实省委十二届六次全会决策部署和省委书记李希同志肇庆调研讲话要求，落实好“1+1+9”工作部署，按照省委、省政府各项工作安排和市委十二届八次全会确定的目标任务，在市委的坚强领导下，统筹推进“五位一体”总体布局，协调推进“四个全面”战略布局，坚持稳中求进工作总基调，坚持新发展理念，坚持推动高质量发展，坚持以供给侧结构性改革为主线，坚持全面深化改革开放，统筹做好稳增长、促改革、调结构、惠民生、防风险、保稳定各项工作，进一步稳就业、稳金融、稳外贸、稳外资、稳投资、稳预期，解放思想、东融西联、产业强市、实干兴肇，举全市之力推进粤港澳大湾区建设，打好三大攻坚战，持之以恒推进产业、乡村、旅游三大振兴，持之以恒推进工业发展“366”、创新驱动“1133”、现代农业“611”三大工程，持之以恒开展全国文明城市、国家创新型城市、国家生态文明建设示范市三大创建，构建“一核三极一区”区域协调发展新格局，聚焦新区崛起府城复兴，保持经济持续健康发展和社会大局稳定，加快建设粤港澳大湾区连接大西南枢纽门户城市，为全面建成小康社会收官打下决定性基础，以优异成绩庆祝中华人民共和国成立70周年。

　　全市经济社会发展主要预期目标是：地区生产总值增长6.5%，规模以上工业增加值增长7.7%，固定资产投资增长10%，社会消费品零售总额增长9%，外贸进出口总额增长3%，实际吸收外资增长5%，一般公共预算收入增长10%，R&D占GDP比重提高到1.9%，居民人均可支配收入增长与经济增长基本同步，居民消费价格指数涨幅3%以内，城镇登记失业率3%以内，节能减排和空气质量改善目标控制在省下达计划以内。

　　重点抓好以下十个方面工作：

　　一、全力以赴扶持实体经济，保持经济稳中向好态势

　　大力支持民营经济发展。抢抓国家和省新一轮促进民营经济发展机遇，制定我市“民营经济十条”，推动民营经济大发展。保持稳定实体经济系列政策连续性，落实减税降费措施，保持市级零收费。强化民营企业法律保障，改进环保、消防、气象等执法方式。完善政银企常态化对接机制，依托“粤信融”平台拓展肇庆分平台功能，争取在全省率先建成线上金融服务载体，协助企业解决融资难融资贵问题。开展就业对接行动，年内举办招聘会不少于150场，协助企业解决用工难问题。持续优化“书记市长强企直通车”，建立万企诉求大平台和处理中心，精准解决企业诉求，着力构建“亲”“清”新型政商关系。

　　持续扩大有效投资。计划安排市重点项目269项，年度计划投资约666亿元，投资额增长17%。大力推动新一轮基础设施补短板，推进“三核九环”电网目标网架、信息网络、市政和水利工程等项目建设。力争落实10亿元产业投资引导基金，发挥政府投资对社会投资的引导和带动作用，加快工业项目落户建设。谋划一批重大项目纳入国家和省规划项目库。

　　有效稳定外贸外资。支持汽车配件、电子信息和传统产业等出口企业做大做强。对接广东自贸试验区，推广应用国际贸易“单一窗口”，壮大发展跨境电商、外贸综合服务、市场采购贸易等新业态新模式，加快肇庆新区综合保税区、四会保税物流中心（B型）、肇庆跨境电商综合试验区申报建设。大力实施省、市外资政策，加快外资企业落地建设，推动现有外资企业增资扩产。

　　坚决打好防范化解重大风险攻坚战。加强政府债务限额管理，落实隐性债务化解计划，确保债务风险稳定可控，持续有效发挥大融资效应。全面完成农合机构改制组建农商行工作。引进发展金融机构4家，新增上市（挂牌）企业3家，力争金融业增加值增长10%以上。优化重组水务、交通、金控、城投、文旅五大投资集团，做强做优国有资产。探索城乡统一的建设用地市场机制，促进房地产市场平稳健康发展。

　　二、深入实施“366”工程和“1133”工程，推动经济高质量发展

　　持续开展“三个年”活动。坚持项目不动工，一切都落空；项目不投产，一切都白讲的理念。突出抓好“项目落地年”，全面推广“双容双承诺”制，推动企业投资项目分类管理和落地便利化改革，确保小鹏汽车等项目投产见效益，新增落地开工产业项目100个以上、年度计划投资178亿元以上，竣工投产项目65个、年度计划投资65.2亿元。一一甄别2015年以来签约项目，并形成有效项目清单，实行“一个项目、一个主责人”定期会商，切实提高履约率和资金到位率。精准推进“招商引资年”，坚持数量与质量并重，聚焦深圳、北京、广州、长三角等地区，探索实施重大项目引进“绿色通道”，深化以商引商、产业链招商，力争引进投资超百亿项目3个以上、超10亿项目20个以上。借助浙江万洋、深圳平谦国际等招商平台，引进一大批中小微企业。深入开展“园区建设年”，引进民营资本加快园区开发和通用厂房建设，新增开发和盘活园区用地不少于10平方公里，新建成及盘活改造通用厂房70万平方米。全面铺开低效园区整改转化，加快广宁华南再生资源产业园等改造升级。

　　加快构建现代产业体系。深入推进工业发展“366”工程，推动新能源汽车、先进装备制造、节能环保产业产值分别达到440亿元、270亿元、190亿元，力争新增年主营业务收入超百亿元工业企业1家、超亿元工业企业40家左右，新增规上工业企业150家以上。对接省数字经济发展规划，加快肇庆高速高质网络服务建设，提前布局5G网络。深入推进创新驱动发展“1133”工程，新增高企150家，加快在建拟建4所本科院校建设，力争再引进1－2所本科院校，谋划建设广东省实验室，确保建成20家新型研发机构、25家高水平科技企业孵化器及众创空间。主动参与大湾区国际科技创新中心建设，打造西江创新产业走廊。紧抓省扩大高新技术开发区布局机遇，力争省级西江高新区、高要高新区获批，组织申报省级四会高新区。大力实施“西江人才计划”和“百千万”人才引育工程，新引进一批高层次人才。

　　实施国家高新区综合评价争先进位行动。高标准建设国家自主创新示范区和国家科技成果转移转化示范区，力争新增高企50家，尽快实现园区高企占全市高企比例和占全区企业比例“双过半”。加快创新创业科学园等双创、孵化平台建设，力争孵化器达到8家、孵化面积达到50万平方米，再新增1家国家级孵化器，高新技术企业产值占规上工业总产值超过60%。

　　三、深化改革开放，举全市之力推进粤港澳大湾区建设

　　树牢大湾区意识和大湾区标准。深度对接“港澳所需”“广东所能”“湾区所向”“肇庆所要”，强化主角意识，用好改革开放关键一招，宏观上对标最高最好最优，要有放眼世界的眼光；微观上做到落细落小落具体，要有亲力亲为的作风。处理好谋定而后动和看准就动的关系，长短结合谋划一批对接大湾区项目，尤其推动政策机制“软联通”，做到先上车找到座位，争当大湾区新秀。

　　深入构建大湾区1小时交通圈。配合做好珠三角新干线机场规划及周边综合配套设施建设，力争机场西部高速（肇明高速）立项建设。谋划推动深江肇高铁、珠江肇高铁、广湛客专、柳广铁路、广茂铁路改线等项目。协调推动肇庆站至佛山西站城轨公交化，增加肇庆往返深圳和增开肇庆直通香港高铁班次，增开怀集始发经由广宁至深圳和珠海高铁班次。深入推进广佛肇一体化建设，抓好对接佛山路网工程项目建设，确保东进大道三期建设、汕湛高速肇庆段建成通车，力争广佛肇高速二期大旺至广州石井（肇庆段）完成主体工程，加快推进怀阳高速肇庆段及肇庆大桥扩建、国省道升级改造。参与粤港澳港口国际合作，积极发展“多式联运”，力争将肇庆新港打造成为珠江内河枢纽港，打造大湾区现代物流基地和配送中心。

　　加强产业共建。积极建设粤港澳大湾区具有重大影响力的合作平台，推进与深圳、广州等城市的多层次、系统性产业合作，参与搭建大湾区“一带一路”共用项目库。发挥好新区的核心载体作用，谋划建设大西南－大湾区科技产业园，推动大湾区和大西南共建产业创新平台。加快建设肇庆（怀集）绿色农副产品集散基地，打造种养、加工、冷链配送的全产业链。支持粤桂合作特别试验区建设，发挥好粤桂产业协作平台作用。

　　全面开展民生领域交流合作。参与粤港澳人才合作示范区建设，打造港澳青年创新创业基地，力争港澳高校在我市设立研发和附属机构，建设大湾区应用型高等教育基地。着力建设大湾区优质生活圈和生活基地，加强与港澳医疗合作，支持港澳优质医疗资源在肇庆以多种形式设立服务机构。聚焦大健康产业，谋划建设大湾区（德庆）南药健康产业基地。强化文旅交流合作，继续办好粤港澳青年厨艺大赛等活动。

　　四、加快构建“一核三极一区”区域协调发展新格局，着力解决发展不平衡不协调问题

　　优化发展城市核心区。统筹推进端州、鼎湖、肇庆新区和高要城区深度一体化融合发展，加快环城快速路建设，着重发展都市型经济，提高城市首位度。加快端州－高要城区组团老城更新、跨江融合，着力在综合城市功能、现代服务业、创新型产业和营商环境等方面出新出彩。巩固提升鼎湖－肇庆新区组团同规划、同建设、同管理、同招商，在社会治理和产业建设等方面深化改革，形成可复制可推广经验。

　　建设产业引领重要增长极。肇庆高新区、高要、四会突出产业引擎功能，依托国家级高新区、省级产业园区、专业园区等重要平台，重点发展新能源汽车、先进装备制造、战略性新兴产业和汽车零配件，推动产业高端化发展，加速千亿产业集聚。完善“一区多园”机制，带动山区县工业园区发展，加快形成增长极与功能区优势互补、差异化发展的产业链条。

　　推动生态发展区绿色崛起。发展生态产业化和产业生态化为主的生态经济，推进广宁、德庆、封开、怀集发展特色支柱产业，集中财力做强核心园区，研究制定对口帮扶机制，推动高新区产业与广宁、怀集互动，新区产业与德庆、封开互动，促进区域协调发展。加快县城扩容提质，发挥城镇对农村地区的服务与辐射功能，完成广宁南街、封开江口、怀集怀城和闸岗“镇改街”，提升基本公共服务均等化水平。

　　五、全力加快肇庆新区崛起，建设彰显中国特色、展现岭南文化的现代化新区和高质量发展引擎

　　对标国内外最高最好最优规划建设。围绕建设成为国家级新区、粤港澳大湾区连接大西南枢纽门户城市核心载体、大湾区高质量发展新引擎“三个目标”，推进总体规划扩容修编，提升规划设计品质。突出高品质市政设施、高端公共服务、高质量产业项目、高效政务环境、高层次人才“五个引领”，统筹开发地上地下立体空间，优化“依山傍湖绕湿地，拥江抱峡望砚洲”生态格局，强化产业新城、创新高地、智慧城市、生态家园功能建设。

　　大思路大手笔大格局推进基础设施建设。聚焦8平方公里核心区，基本建成地下综合管廊，完成长利湖等水系工程，加快完善污水处理厂及管网等市政设施，精心打磨“一路一树一灯”城市景观，促进中山三院附属肇庆医院、湿地景观酒店等公共服务设施投入使用。做好体育中心、东站综合体运营。抓好整村搬迁安置，加快康盛花园等安置小区规划建设。

　　培育壮大战略性新兴产业。新引进超百亿项目2个，新增落地开工项目15个，产业项目完成投资超80亿元，力促项目落地动工见效。大力发展数字经济，加快推进中国电科肇庆产业园等新兴产业项目，建设数字经济高地。大力发展门户经济，加快“西江明珠”、中国民商理事会总部大楼、贵州企业（粤港澳大湾区）联合总部基地等总部工程建设，推动创新创业、总部办公及人口快速集聚。加快推进粤港澳大湾区生态科技产业园、孵化创新园建设。确保肇庆新港上半年动工。力争获批国家级综合保税区。

　　六、加强城市规划建设管理，焕发高品质新都市新活力

　　高起点规划提升城市魅力。出台实施新一版城市总体规划，高标准持续开展各类规划编制，科学控规重点区域特别是星湖、北岭山、鼎湖山、羚羊峡周边的城市景观、建筑高度和天际线。引进社会资本大手笔做好府城保护开发这篇大文章，精心实施古城墙修缮保护工程，完成府城公园西段建设，延续千年文脉，打造岭南文化新地标。积极申报中国人居环境综合奖。

　　精准创文推动城市“微改造”。推动创文与日常工作深度融合，按照“一年一主题”统筹落实“新十大提升计划”，做到每月一暗访一研判。持续抓好民生微实事，全面推动老旧小区、集贸市场、背街小巷整治等硬件工程收尾工作，大力推行垃圾分类，加快社区标准化建设，积极创建全国无障碍建设城市，确保年度测评成绩进入全国前列。启动一批城中村整村改造项目，完成端州“瓶颈路”建设收尾工程，完成市汽车客运总站搬迁，加快公共停车场及智慧停车项目建设。纵深推进“四级联创”，完善基层公共文化设施，提高城乡文明程度。

　　旅游振兴壮大都市型经济。出台实施“旅游振兴十条”，全力推进星湖景区创5A，大力推动旅游景区创A、酒店评星和旧景区设施盘活，启动创建国家全域旅游示范市。谋划一批旅游新项目，建设一批文化精品，将《青天之端》打造成为全国廉政文化样板。谋划西江游，促进西江沿线旅游资源串珠成链，打造千里文化生态旅游大走廊。加快建设华侨城卡乐文化旅游科技产业小镇等龙头项目，持续办好肇庆国际马拉松赛、全国田径大奖赛、全国体操锦标赛和粤港澳大湾区（肇庆）光影艺术节等品牌活动。争取将“请到广东过大年”主会场长期落户肇庆，发展具有肇庆民俗特色的喜庆节庆文化。

　　七、突出抓好“611”工程，深入推进乡村振兴

　　坚决打赢精准脱贫攻坚战。全面落实“两不愁、三保障”等扶贫政策，坚持一户多策、一人一办法，拓宽贫困户增收致富渠道，力争全市建档立卡贫困人口全部实现预脱贫。开展精准扶贫“回头看”，研究解决收入水平略高于建档立卡贫困户群体缺乏政策支持等新问题。加强扶贫资金使用与监督监管。开展好对口桂林、贺州两市四县扶贫协作。

　　大力发展富民兴村产业。深入开展6大百亿元产业集群创建行动，探索设立农业供给侧结构性改革基金。加快推进已获批的4个省级现代农业产业园建设，力争新增4个省级现代农业产业园、2个省级农业科技园，培育一批省级“一村一品、一镇一业”示范镇村。大力引进国内外品牌农业龙头企业，新增一批农业龙头企业、农民合作示范社、示范性家庭农场，推动行政村电商全覆盖。加快高要全国农村一二三产业融合发展先导区、德庆国家农村产业融合发展试点示范县建设，打造高要“河乐水”等一批乡村旅游示范区。全面落实粮食安全责任制。深化农村综合改革，推动“两权”抵押贷款试点和普惠金融“村村通”纵深发展。全力推进新乡贤返乡、“粤菜师傅”培训、“千企帮千村”等工作，开展乡村游和农家乐，多点带动农民增收。

　　加快实施“百村示范、千村整治”工程。全面完成村庄规划编制，建立农村违法建设用地处置预警机制。持续深入推进全域农村人居环境整治，确保全市村庄基本完成“三清三拆三整治”、80%以上达到干净整洁村标准。高标准建设省际廊道美丽乡村示范区，完善与广西接壤地区11个镇规划设计，全面启动示范项目建设。每个县（市、区）再启动创建1个以上乡村振兴示范镇“书记项目”。加快省级新农村连片示范工程，巩固提升111个省定贫困村新农村示范村建设。持续推进“厕所革命”、生活污水处理设施、“四好农村路”、电网改造、农村饮用水安全等建设。

　　　八、全力创建国家生态文明建设示范市，走绿色发展路子

　　坚决打好污染防治攻坚战。全面落实中央和省环保督察问题整改要求，按时保质完成年度整改任务。全力打好蓝天保卫战，重点做好污染源头管控，全面关停一批小散污企业，成立陶瓷产业退出转型基金，抓好陶瓷产业引导退出工作，推动空气质量持续好转。全力打好碧水攻坚战，加快互联网+河（湖）长信息化建设，加快西江干流整治和中小河流治理，开展河湖清“四乱”专项整治，推进羚山涌和石咀涌城市黑臭水体综合整治，持续改善星湖水质。大力解决端州城区污水处理历史欠账，全面启动端州城区雨污分流和管网新建改建。整县推进污水处理设施及配套管网建设，全面建设63座镇级污水处理设施。完成县级以上饮用水源保护区“划、立、治”重点任务，力争省考以上断面水质优良率达到考核标准。全力打好净土防御战，开展受污染耕地和工业污染地块治理修复工作。依法严厉打击非法转移、倾倒、处置固体废物行为，规划并控制好固废产业。

　　统筹推进“绿富同兴”。坚持“三个绝不”，加强招商项目环保审核，抓好节能减排，确保肇庆环保能源发电项目点火运行。落实桉树林分改造任务，建设一批森林小镇和绿美古树乡村，加快广宁林下经济发展示范县建设，加强自然保护区管理，积极创建全国绿化模范城市和国家生态园林城市。抓好土地例行督察整改和违法违规用地整治，加快创建全国国土资源节约集约模范市。大力推进“三旧”改造和闲置土地清理。继续抓好瓷土矿资源开发秩序专项整治。加强河砂供应链管理，探索“统采统销”模式。

　　积极创建国家生态文明建设示范市。健全创建工作机制，力争年内36项指标全面达标。积极创建一批市级生态示范村，力争创建1个国家生态文明建设示范县。持续加大生态系统保护力度，实施重要生态系统保护和修复工程。

　　九、坚持以人民为中心，切实保障和改善民生

　　做好就业、社保、住房等基本民生。城镇新增就业4.3万人。强化社会政策兜底保障，持续加大民政等民生投入，按时足额发放社保待遇。完善医保按病种分值付费配套制度。加强社区服务能力建设，探索推进社区居家养老“大配餐”、医养结合试点工作。构建完善退役军人服务体系。完成农村危房改造1673户。鼓励支持老旧住宅楼加建电梯试点工作，加强小区物业管理。

　　改善教育、医疗、公共交通等热点民生。全面实施教育提升发展十大行动计划，加快推进优质教育资源下县（市、区），推进集团化办学，增加优质学位供应，有效提升教育质量，将更多学生送进国内外重点大学。落实好教师待遇“两相当”政策。支持肇庆学院实施粤东西北高校振兴计划。抓好老年教育。加快创建全国科普示范市，提升全民科学素养。推进卫生健康事业发展十大行动计划，加快4间中心卫生院升级为县级第二人民医院，推进13个县级医院升级建设项目，深化医联体等综合卫生改革。逐步提高乡村医生待遇，建设好基层医疗队伍。加大重大疫情防控力度。继续实施公共交通畅通工程，加快推进端州、鼎湖、高要公交一体化。

　　营造共建共治共享社会治理格局。推动扫黑除恶专项斗争向纵深发展，全方位打击涉黑涉恶势力，深入开展扫黄打非专项行动，加快建设“智慧新警务”“雪亮工程”，积极创建全国社会治安综合治理优秀市，全面建设平安肇庆。持续推进全民禁毒工程。进一步畅通信访渠道，推进首批市级社会治理实践创新项目实施，推动社会治理重心向基层下移。加强安全生产监管、应急管理、防灾减灾等工作，强化党政“一岗双责”和企业安全主体责任，确保全市安全生产形势总体平稳。扎实创建省级食品安全示范城市。

　　进一步加强国防动员、后备力量建设，全面启动全国“双拥”模范城创建。扎实推进与鸡西市对口合作。全力做好第四次全国经济普查。继续做好民族、宗教、革命老区、外事、侨务、港澳、对台、人防、地方志、档案、气象、水文、地震、妇女儿童、青少年、老龄、残疾人、慈善等工作。在办好省民生实事的同时，继续办好市十件惠民实事。

　　　十、深化政府职能改革，全面优化营商环境

　　加强政府系统政治建设。严明政治纪律和政治规矩，落实全面从严治党主体责任，扎实开展“不忘初心、牢记使命”主题教育，坚持“第一议题”制度，促进全市政府系统树牢“四个意识”，坚定“四个自信”，坚决做到“两个维护”，自觉在思想上政治上行动上同以习近平同志为核心的党中央保持高度一致。

　　加强法治政府建设。严格遵守宪法法律，依法接受人大监督，自觉接受政协民主监督，定期向人大及其常委会报告工作和向政协通报情况，认真办理人大代表建议和政协提案，继续办好“市长直通车”，广泛听取民主党派、各人民团体、各界人士意见。加强政府决策合法性审查、专家咨询论证，提高民主决策、科学决策水平。加强政府诚信建设，杜绝“新官不理旧账”。全面推进政务公开，充分发挥审计监督和保障作用，主动接受巡视巡察监督。

　　加强效率政府建设。全力推进机构改革，强化权责统一，加快构建上下贯通、执行有力的机构职能体系。深化强县放权改革，推动市级权限应放尽放。要做到“干”字当头，持续推进牵头单位主责制、“一线工作法”、重大事项会商制，坚决破除文来文往、以会代干、议而不决，不断提高抓落实水平。持续优化无纸化办公系统，全面推进数字化移动协同办公系统应用。加强调查研究和统计分析，提高在复杂形势下把握宏观经济的能力。

　　加强“数字政府”综合改革。按照应进必进要求，加快一体化在线政务服务平台建设，推进更多公共服务汇聚到“粤省事·肇庆行”，持续深化“一门受理、一网通办、一次办成”。全面清理各类审批证明材料，进一步压缩工程建设审批和不动产登记办理时间。完善并落实好“双容双承诺”产业项目直接落地制度改革。

　　加强廉洁政府建设。严格执行中央八项规定及其实施细则精神，严防“四风”问题反弹回潮，从严整治“庸懒散奢”等不良风气。扎实抓好模范机关创建活动。树立过“紧日子”思想，厉行节约。严格政府投资项目概算和预算管理，完善全流程电子化交易，严控围标、串标等违法违规行为。

　　各位代表，新挑战新机遇，新肇庆新希望！让我们更加紧密地团结在以习近平同志为核心的党中央周围，高举习近平新时代中国特色社会主义思想伟大旗帜，按照省委、省政府决策部署，在市委的坚强领导下，不忘初心、牢记使命，闻鸡起舞、日夜兼程、风雨无阻，解放思想、实干兴肇，为全面建成小康社会、加快建设粤港澳大湾区连接大西南枢纽门户城市而努力奋斗！为广东实现“四个走在全国前列”、当好“两个重要窗口”作出肇庆新的更大贡献！以优异的成绩迎接中华人民共和国成立70周年！
